# Oxazolidinone Antibiotics: Chemical, Biological and Analytical Aspects

**DOI:** 10.3390/molecules26144280

**Published:** 2021-07-14

**Authors:** Claudia Foti, Anna Piperno, Angela Scala, Ottavia Giuffrè

**Affiliations:** Department of Chemical, Biological, Pharmaceutical and Environmental Sciences, University of Messina, Viale F. Stagno d’Alcontres 31, 98166 Messina, Italy; cfoti@unime.it (C.F.); apiperno@unime.it (A.P.); ascala@unime.it (A.S.)

**Keywords:** oxazolidinone antibiotics, Linezolid, TBI-233, analytical determination, antibiotic resistance

## Abstract

This review covers the main aspects concerning the chemistry, the biological activity and the analytical determination of oxazolidinones, the only new class of synthetic antibiotics advanced in clinical use over the past 50 years. They are characterized by a chemical structure including the oxazolidone ring with the *S* configuration of substituent at C5, the acylaminomethyl group linked to C5 and the *N*-aryl substituent. The synthesis of oxazolidinones has gained increasing interest due to their unique mechanism of action that assures high antibiotic efficiency and low susceptibility to resistance mechanisms. Here, the main features of oxazolidinone antibiotics licensed or under development, such as Linezolid, Sutezolid, Eperezolid, Radezolid, Contezolid, Posizolid, Tedizolid, Delpazolid and TBI-223, are discussed. As they are protein synthesis inhibitors active against a wide spectrum of multidrug-resistant Gram-positive bacteria, their biological activity is carefully analyzed, together with the drug delivery systems recently developed to overcome the poor oxazolidinone water solubility. Finally, the most employed analytical techniques for oxazolidinone determination in different matrices, such as biological fluids, tissues, drugs and natural waters, are reviewed. Most are based on HPLC (High Performance Liquid Chromatography) coupled with UV-Vis or mass spectrometer detectors, but, to a lesser extent are also based on spectrofluorimetry or voltammetry.

## 1. Introduction

The word antibiotic was first introduced in 1941 by Selman Waksman to indicate any small molecule produced by a microbe capable of counteracting the growth of other microbes [1,2]. In the years between 1940 and 1960, a period known as the antibiotic golden age, penicillin, streptomycin, tetracycline and chloramphenicol were developed [1,2]. However, the evolution of bacterial resistance has made these antibiotics and many other successors largely ineffective [1].

According to the WHO (World Health Organization) definition, antimicrobial resistance refers to change in microorganisms (viruses, bacteria, parasites and fungi) which make the drugs used to treat the infections they have caused ineffective [3]. Such resistance to a wide variety of infectious agents is a major global health concern and the rapid spread of multidrug-resistant bacteria is alarming [4,5]. In recent years, bacterial resistance to first-line drugs has reached very high percentages and, unfortunately, resistance to second- and third-line drugs has also become quite common. Of particular concern are the multidrug-resistant and cross-resistant strains [5], including methicillin-resistant Staphylococcus aureus (MRSA), which is responsible for traditional treatment failure, high mortality rate and prolonged hospital stays. 

The European Antimicrobial Resistance Surveillance Network (EARS-Net) provides data on antimicrobial resistance in the EU and European Economic Area (EEA), estimating that there were 671,689 cases of infections with selected antibiotic-resistant bacteria in 2015. This resulted in 33,110 attributable deaths, corresponding to an incidence of 131 infections and a mortality rate of 6.44 deaths per 100,000 inhabitants [6]. More specifically, it was found that antibiotic-resistant bacterial infections occur predominantly in hospitals and other health services. Among EU and EEA countries, Italy and Greece had a significantly higher percentage of carbapenem-resistant or colistin-resistant bacteria. In order to counter this alarming data, Italy published the 2017–2024 National Action Plan on Antimicrobial Resistance, including objectives for reducing the use of antibiotics both in human and veterinary fields and care-related surveillance, prevention and control. In this context, scientific research and innovation are at the basis of international law enforcement policies on antimicrobial resistance (AMR) and represent the fundamental conditions for identifying and transferring new effective tools to counter this phenomenon [6]. 

By 2014, data on multidrug-resistant tuberculosis (MDR-TB) were made available from 153 countries, accounting for 96% of the world population and cases of incident tuberculosis (TB) [7]. Worldwide, it was estimated that, on average, 3.3% of newly diagnosed TB patients and 20.1% of those with previously treated TB have MDR-TB [7,8]. 

**Figure 1:**
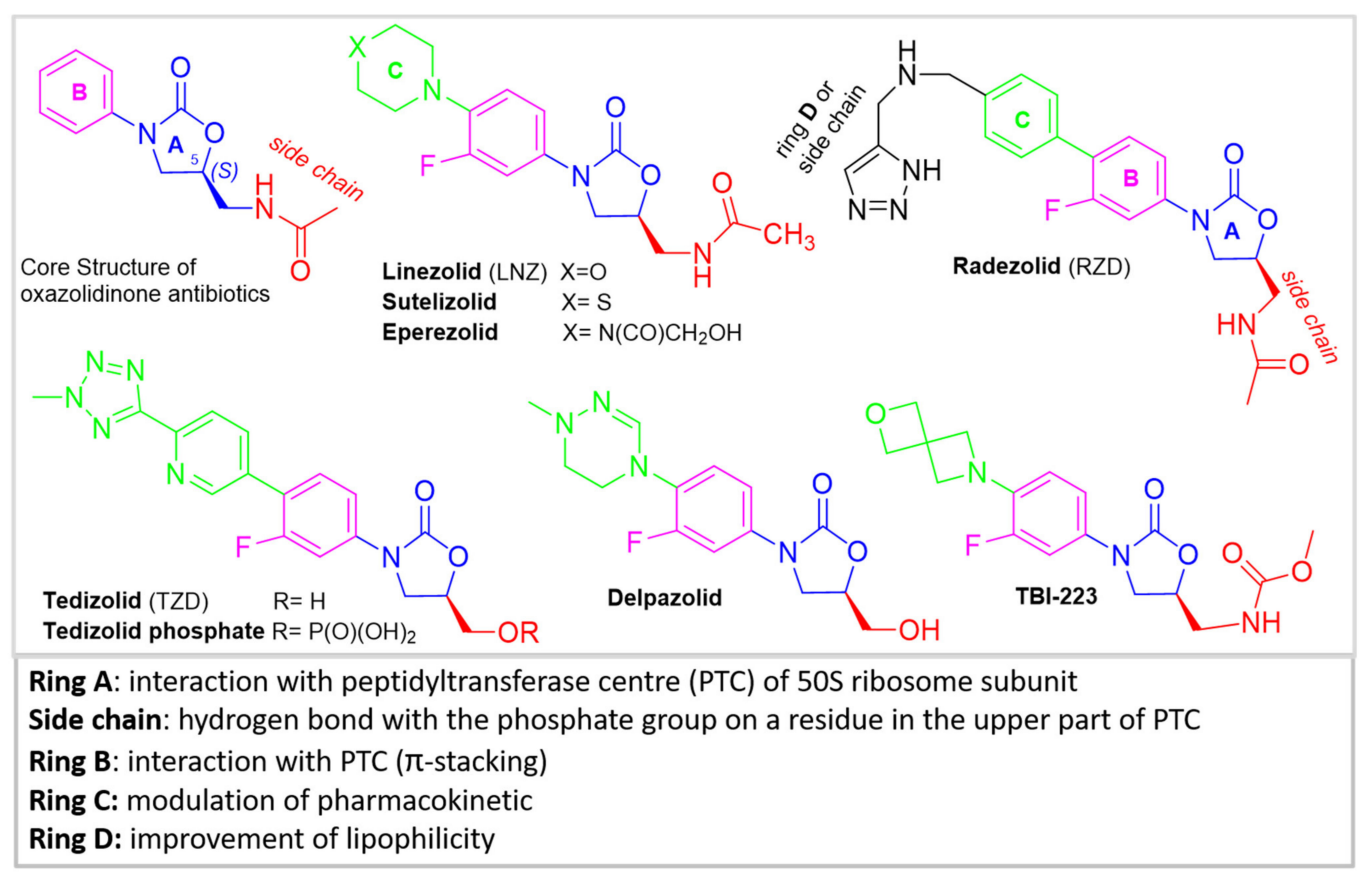
Core structure of oxazolidinone antibiotics and molecular structures of main derivatives (Linezolid, Sutezolid, Eperezolid, Delpazolid, Tedizolid, Tedizolid phosphate, Radezolid and TBI-223). Brief description of chemical groups required for the binding to peptidyltransferase center (PTC) and chemical groups that improve the pharmacokinetics.

Research in this field is primarily aimed at the development of new technologies (new antibiotic therapies, new vaccines), understanding the ways in which antibiotic-resistant microorganisms emerge and spread, formulating strategies for the responsible use of antibiotics and reducing the risk of infections in healthcare [6,9]. Therefore, the search for new antibiotics as well as the development of new strategies to delay resistance to existing ones has become essential [1]. Until the 1980s, pharmaceutical research and industry provided antibacterial agents, characterized by a new mode of action, capable of evading resistance to previous drugs. From the 1990s onwards, new drugs, such as oxazolidinones and lipopeptides, were introduced for the treatment of infections caused by multiresistant Gram-positive/Gram-negative bacteria. Oxazolidinones are a recent class of synthetic antibiotics with a chemical structure characterized by a basic nucleus of 2-oxazolidone (Figure 1) active against a wide spectrum of multidrug-resistant Gram-positive bacteria (GPB), namely vancomycin-resistant Enterococcus (VRE), MRSA and Mycobacterium tuberculosis (Mtb) [10]. Oxazolidinones bind to the 50S ribosomal subunit, inhibiting the biosynthesis of bacterial proteins [10]. The first oxazolidinone clinically available was Linezolid (LNZ), discovered in 1996 and approved in 2000 for clinical use by the FDA (U.S. Food and Drug Administration) [11]. LNZ is widely employed for GPB infections and it is considered an efficient drug for surgical infections [11,12] and in the treatment of drug-resistant pulmonary infections and MDR-TB infections. Among oxazolidinones, only LNZ and Tedizolid are clinically approved for MDR-TB infections. Tedizolid (TZD) belongs to the second generation of oxazolidinones and is also indicated for the treatment of skin infections [13]. Radezolid (RZD), belonging to the biaryl oxazolidinone family, is effective against resistant LNZ strains. Although clinical trials into community-acquired pneumonia and into skin and soft tissue infections have concluded, studies on its acceptability are not yet finished [14]. In the field of treating MDR-TB infections, many efforts have been made to discover the next generation of oxazolidinones having better antibacterial efficacy and fewer adverse effects. Recently, several oxazolidinone analogs have been developed at well-known pharmaceutical companies, some of which have been found to be suitable for treating MDR-TB [15]. Among them, Sutezolid (STD), the next analogue after LNZ, TZD and RZD, has proved to be promising. The molecular structure of STD includes three rings, i.e., oxazolidin-2-one with a (methyl)acetamide substituent, fluorophenyl and thiomorpholine (Figure 1). The stereogenic center at position C-5 of the oxazolidin-2-one ring is related to its antibacterial activity [16]. STD is in clinical trial for the treatments of MDR-TB. 

Gram-negative bacteria (GNB), such as Enterobacter and Escherichia coli, are responsible for severe infections, such as pneumonias, surgical infections, urinary tract infections and meningitis. Recently there has also been a worrying increase in MDR across several species of GNB and strains resistant to many of the available antibiotics [17]. As outlined in a recent report by the Centers for Disease Control and Prevention (CDC), MDR GNB were listed as one of the most urgent threats to be addressed [17,18]. The outer membrane (OM) of GNB and the associated proteins outside their cytoplasmic membrane make them harder to reach than GPB [19]. OM constitutes a powerful barrier that hinders entry of the drug due to the outer flap that prevents passive diffusion, the channel proteins that limit the size and properties of the solutes and the efflux pumps that carry the compounds outside the cytoplasmic membrane. Such barriers greatly reduce the antibacterial activity of drugs against GNB. Therefore, to be effective against GNB, antibacterials must be characterized by a low molecular weight, a rather high polarity and a charged character. The oxazolidinone group holds great potential against GNB. A decisive feature of this class is that resistance to LNZ, which is rare in GPB and practically absent in GNB, can be largely avoided by structural changes of LNZ. In order to predict clinical success for new oxazolidinones, it is possible to make their preclinical evaluation for pharmacokinetic/pharmacodynamic (PK/PD) parameters by considering data on the structure/activity relationship (SAR) related to antibacterial activities [17].

Moreover, the PK/PD features of oxazolidinones can be improved by exploiting appropriate drug delivery systems that allow us, by drug incorporation or covalent conjugation, to overcome the main hindrances related to the clinical use of oxazolidinones, including poor water solubility and adverse systemic effects.

A series of new compounds, including bis-oxazolidinones, have been synthesized and tested in vitro for antibacterial activities as potential antitubercular agents [20]. Due to worrying growth around the world of bacterial resistance to antibiotics, their analytical determination in biological fluids has gained increasing interest. Analytical methods for oxazolidinone determination are also relevant for environmental applications, since antibiotics are released into natural waters and accumulate in the soil, threatening the health of living beings [21]. 

## 2. Chemistry of Oxazolidinone Antibiotics

Oxazolidinones are a fully synthetic class of bacteriostatic agents that find no congeners in natural product compounds and were the first synthesized at E.I. du Pont de Nemours & Co. in the mid-1980s and successively implemented by Pharmacia (now Pfizer Inc., New York, NY, USA). Their historical development has been discussed in different literature reviews [22,23]. The first initial SAR studies identified the first non-toxic oxazolidinones (e.g., LNZ and Eperezolid) and the essential structural factors required for their biological activity (Figure 1). Specifically, the pharmacophoric core includes the oxazolidone ring with the *S* configuration of substituent at C5 and the acylaminomethyl group linked to C5 and the *N*-aryl substituent (ring B). The meta-fluoro substitution on B ring increases the biological activity, whereas the para-substitution expands the antibacterial spectrum [24]. The mechanism of action was elucidated using the X-ray co-crystal structures of LNZ bound to 50S ribosomal subunit, reported in 2008 by two independent research groups [25,26]. 

Oxazolidinones inhibit protein synthesis by interaction with the A-site pocket of the 50S subunit at the peptidyl transferase center (PTC). This interaction affects the binding and/or positioning of the initiator-tRNA and prevents the binding of tRNA at the A site, thereby preventing the translation sequence. 

Both LNZ and Eperezolid emerged from preclinical tests as potential drug candidates showing almost identical MIC (lowest concentration of a compound/antibiotic at which no growth is observed) values, antibacterial spectrum and pharmacokinetic parameters. LNZ was selected for further development for its prolonged half-life in humans; these data emerged after LNZ and Eperezolid Phase I clinical studies. 

**Scheme 1:**
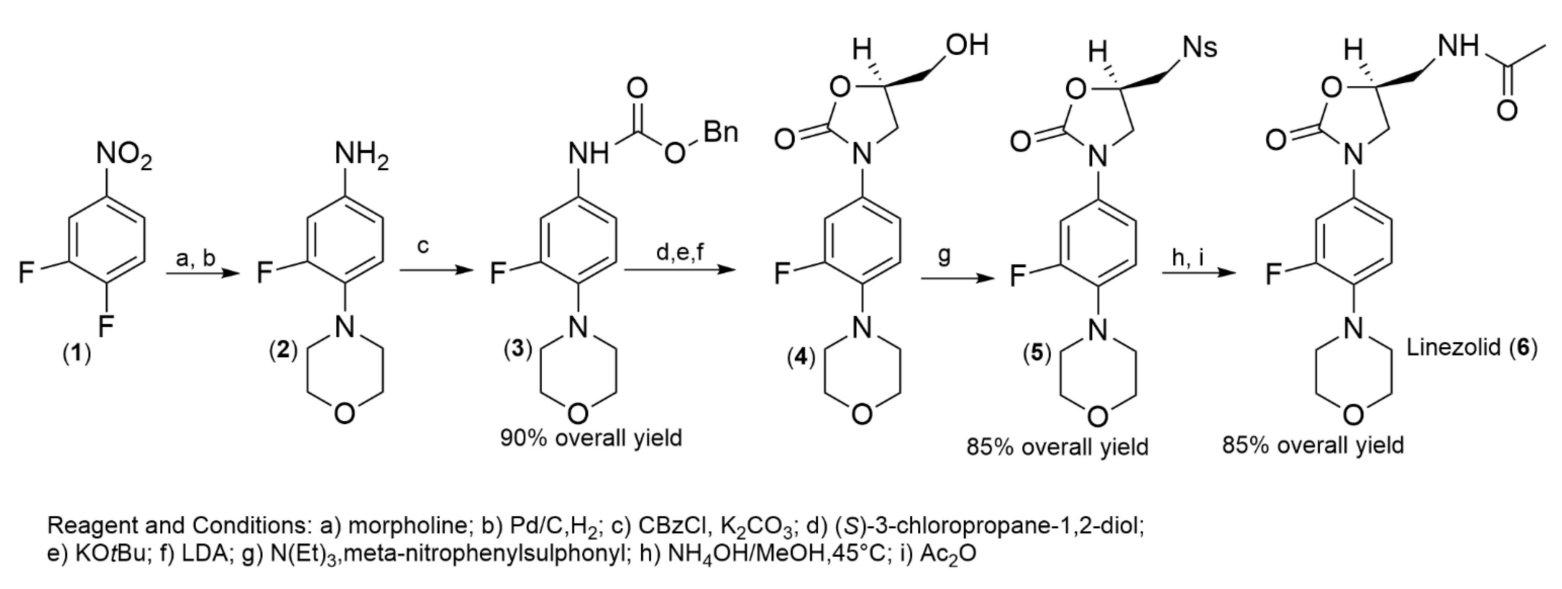
Synthetic strategy for large-scale LNZ synthesis.

The chemical strategy for the large-scale synthesis of LNZ is described in Scheme 1 [22].

Therapeutic treatments based on LNZ improved the outcomes of several drug-resistant infections, including TB; however, long-term side effects such as reversible myelosuppression, potentially irreversible optic neuropathy and peripheral neuropathy are often correlated to its prolonged administration [27]. To overcome these critical issues, different derivatives with improved safety and tolerability were approved by local regulatory agencies or are under development for diseases that require long-term therapy. The main representative oxazolidinone antibiotics licensed or under development together with their main features are described in Table 1.

## 3. Analytical Determination

The phenomenon of bacterial resistance to antibiotics is constantly increasing in the world and requires methods for their determination in various types of matrices, not only drugs, biological fluids or tissues, but also foods and natural waters. In fact, antibiotics were recently recognized as contaminants of emerging concern (CECs) owing to their wide distribution and persistence in the environment, mostly in wastewaters and in natural water systems. Among the oxazolidinones, LNZ is certainly the one that has attracted the most attention and liquid chromatography, mass spectrometry and electrochemical or spectrophotometric/fluorimetric methods have been developed for its analytical determination.

The most used analytical techniques are certainly based on HPLC (High Performance Liquid Chromatography) methods, mostly coupled with UV-Vis spectrometric detectors. HPLC methods concerning the determination and quantification of LNZ in different matrices (pharmaceutical and biological samples, such as human plasma or serum, urine, but also different kinds of tissues, including brain, eye, pulmonary), since the time of its discovery until 2019, were carefully summarized in a recent review [37]. As pointed out before, most of these are based on the use of UV detectors and differ from each other mainly in the composition of the mobile phase and, to a lesser extent, in the flow rate and the type of column. Depending on different experimental procedures, the limit of detection (LOD) and limit of quantification (LOQ) are extremely variable, ranging between 0.007 ≤ LOD ≤ 0.5 μg/mL and 0.01 ≤ LOQ ≤ 1.6 μg/mL.

In recent years, the increasing use of the mass spectrometer as an HPLC detector has led to the development of analytical methods that take advantage of the greater sensitivity and selectivity of this detector in respect to the UV one. One review [37] summarized the HPLC-MS/MS and Ultra Performance Liquid chromatography–tandem Mass Spectrometer (UPLC-MS/MS) methods developed up to 2019, mostly used to detect LNZ in human plasma. HPLC-MS/MS was also used [38,39] for the simultaneous quantitation of OTB-658 (a novel oxazolidinone anti-tuberculosis agent) and its two major metabolites in monkey blood with good linearity over the range of 10–2000 ng/mL. Very recently, UPLC-MS/MS methods were reported for the determination of TDZ in plasma [40] and in aqueous humor of rabbit, with a LOD of 1.97 ng/mL [38,39]. 

In addition to HPLC methods, other techniques have been used to determine oxazolidinones. Spectrofluorimetric methods were used [41] for LNZ determination in commercial tablets, cream, gel and spray formulations, with a linear response over concentration ranges 0.5–5.0 μg/mL and LOD and LOQ of 110.0 ng/mL and 320.0 ng/mL, respectively. The linear response was extended to the range 20–400 ng/mL using a spectrofluorimetric method [42] based on the use of quinone-based fluorophores to enhance the fluorescence of the molecule. The method was applied to the determination of LNZ in pharmaceutical formulations.

Other methods concern electrochemical techniques, which are interesting for their rapidity, affordability and selectivity. In this area, LNZ was also the oxazolidinone object of most studies [21,43,44,45,46,47]. In [45], the electrochemistry of LNZ was investigated and a method based on differential pulse voltammetry (DPV) was developed for its quantification, using a glass carbon electrode. The method was tested for the quantification of LNZ in pharmaceutical preparation and in urine, with LOD of 50 mg/L and linear response up to 200 mg/L. More recently, the use of new electrode materials, which is one of the main research topics in the field of electrochemistry, has led to the development of new methods [21,43,44,45,46,47]. The most recent are based on the use of graphene oxide–bentonite sodium composite modified electrodes [47], multiwalled carbon nanotubes modified carbon paste electrodes [43,46], unmodified renewable pencil graphite electrodes [44] or boron-doped diamond electrodes pretreated cathodically [21]. All were used for the detection of LNZ in pharmaceutical or biological samples, showing good performance concerning both linear concentration range and LOD. As an example, by using a boron-doped diamond electrode pretreated cathodically for LNZ determination in pharmaceutical formulations [21], calibration curves were linear over concentration ranges of 0.25–6.41 μg/mL, with LOD of 0.05 μg/mL. The best performance obtained by the different instrumental techniques are summarized in Table 2.

## 4. Biological Activity of Oxazolidinones and Delivery Systems

Oxazolidinones are protein synthesis inhibitors active against multidrug-resistant GPB, including MRSA, penicillin-resistant streptococci and vancomycin-resistant enterococci. This peculiar spectrum of action suggests that these compounds inhibit bacterial growth by interfering with protein synthesis with a unique mechanism of action. 

The identification of the binding site and the most likely mode of action took considerable efforts [48] and, in 2008, two studies reporting the X-ray co-crystal structures of LNZ bound to the 50S ribosomal subunit confirmed the precise location of the drug binding site, suggesting a mode of action [25,26]. 

Oxazolidinones bind to and inhibit both bacterial and archaeal ribosomes, but do not interact with human cytoplasmic ribosomes. The mechanism of action differs from all existing protein synthesis inhibitors as the inhibition occurs at a very early stage and involves the binding of N-formylmethionyl-tRNA (tRNAfMet) to the ribosome. The competition with chloramphenicol and lincomycin for binding the 50S subunit indicates that oxazolidinones have close binding sites, even though they do not inhibit peptidyl transferase. They bind to the bacterial 50S ribosomal subunit, at the 23S portion, without interacting with the 30S subunit. Specifically, the main action of oxazolidinones is the binding of the A-site pocket of the 50S subunit at the PTC of the ribosome with consequent inhibition of the initiation complex and translocation of peptidyl-tRNA from A site to P site. 

In detail, LNZ binds to a pocket formed by eight RNA residues, one of which (U2585Ec) is stabilized in a distinct conformation, nonproductive for peptide bond formation. In this way, LNZ affects the binding and/or positioning of the initiator-tRNA and prevents the binding of tRNA at the A site, thereby halting the translation process [24].

Oxazolidinones are used for the treatment of skin and soft tissue infections. Due to the unique mode of action, they have, in principle, a low chance to develop drug resistance. However, owing to the worldwide spread of acquired resistance genes (e.g., cfr, optrA and poxtA), three different oxazolidinone-resistance mechanisms have been characterized so far: the point mutation G2576T in the domain V of 23S rRNA genes (single nucleotide polymorphism); the acquisition of the ribosomal methyltransferase gene designated as “cfr gene”; and mutations in rplD and rplC genes encoding 50S ribosomal proteins L4 and L3, respectively [49,50].

The pharmacokinetic profile of LNZ makes this antibiotic a good choice for the treatment of orthopedic infections such as chronic osteomyelitis, which requires a prolonged administration time. Its oral formulation has an almost 100% bioavailability; moreover, intravenous administration is also available. LNZ is indicated for nosocomial skin infections and it has been included in the WHO’s list of drugs for treating drug-resistant strains of Mtb [51]. 

The adverse effects related to LNZ administration consist of bone marrow suppression in the case of prolonged administration of the antibiotic (more than 2–3 weeks), with related thrombocytopenia, anemia and decreased hemoglobin concentration. However, all blood abnormalities were reversible and returned to baseline values 10–14 days after the end of the treatment. An irreversible peripheral neuropathy may occur in the case of prolonged treatment (more than 6 months) [52]. 

Drug solubility is a critical factor for the clinical development of antibiotics, since the dissolution rate greatly influences their absorption, blood concentration and bioavailability. Since oxazolidinones are poorly water-soluble compounds, the recent advancements in the bio- and nanotechnologies represent a good chance for the development of drug delivery systems (DDS) able to overcome the hindrances related to hydrophobicity. To the best of our knowledge, few classes of nanomaterials have been investigated as carriers for oxazolidinones, including cyclodextrins and biopolymers. Recently, a Tedizolid-hydroxypropyl-cyclodextrin (TZD/HP-CD) inclusion complex was proposed by Cielecka-Piontek and coworkers [53]. The dissolution rate, permeability through an artificial model membrane and the antimicrobial activity of TZD/HP-CD were studied and compared with the parent TZD. The complexation into HP-CD has a positive effect on TZD solubility and membrane permeability; moreover, a delayed-release profile from the nanoformulation and an increased bactericidal effect were observed. Different dissolution profiles of TZD/HP-CD complex compared with free TZD were registered at different pH (double dissolution rate at pH 4.5 and 6.8; triple speed at pH 1.2), pointing out the ability of cyclodextrins to modify TZD release, especially in the gastric environment.

The same authors demonstrated that hydrophilic polymers, such as hydroxypropyl methylcellulose (HPMC) and triblock copolymers based on poly(ethylene oxide) (PEO) and poly(propylene oxide) (PPO) (PEO–PPO–PEO, Pluronic^®^), also significantly influenced the solubility of TZD, increasing and prolonging its release. Nevertheless, the decrease in the permeability hindered further development of the proposed Pluronic-based delivery system [54]. 

Until now, only a few polymeric nanoformulations have been reported. Biodegradable polymers, including poly(lactic-co-glycolic) acid (PLGA), poly(lactic) acid (PLA) and polycaprolactone (PCL), have been proposed for the localized delivery of LNZ to overcome the adverse effects associated with its systemic use.

PLGA and PLGA/PCL electrospun fibers loaded with LNZ were recently evaluated for the treatment of MRSA associated with bone infections, prosthetic infections and soft tissues [55]. The nanofiber formulation, allowing a local application of LNZ, reduced the systemic side effects and guaranteed a sustained release and a prolonged antibacterial activity [56]. 

The use of PLA as local drug delivery system for LNZ was investigated for the direct administration of the drug at the orthopedic implant site, with the aim to reduce the bacterial attachment on wires. The medical implants, namely orthopedic Kirschner wires, were coated by the solvent casting technique with the desired antibiotic–polymer solution, using LNZ at three different concentrations (2.5%, 5% and 10%) to create a local DDS able to efficiently prevent the adhesion of MRSA [57]. 

LNZ-loaded lipid–polymer hybrid nanoparticles (LIN-LPNs) were prepared by nanoprecipitation using lecithin, DSPE-mPEG(2000), cholesterol (as lipidic components) and PLGA (as polymeric core) at a 1:5.7 polymer-to-lipid ratio. The activity of the hybrid nanosystem was evaluated in vitro against intracellular and biofilm-embedded MRSA. LIN-LPNs showed high drug loading (12%) and a sustained release profile (quick release of ~25% payload in the first 4 h, followed by ~70% drug release in 120 h). Enhanced intracellular and anti-biofilm activities were observed with respect to the free drug against these less accessible, antibiotic-resistant pathogens that often cause osteomyelitis chronicity and recurrence [58]. 

LNZ-loaded gelatin nanoparticles were surface-functionalized with mannose (LNZ-Mn-GNPs) for selective targeting to alveolar macrophages as an effective strategy to fight Mtb infection. The primary host of Mtb is the alveolar macrophages where they grow, replicate and then spread through the body; hence, targeting mannose receptors on macrophages can be a useful strategy to treat the disease. The authors reported the in vitro physicochemical characterization and ex vivo hemolytic toxicity evaluation, pointing out the safety and reliability of the formulation [59]. 

As an alternative to traditional nanocarriers, antibiotics can be delivered by siderophores, small-molecule chelators that are produced by bacteria to sequester Fe(III), an essential nutrient required for bacterial growth and virulence. Specifically, the outer membrane proteins of GNB, binding ferric siderophores, provide opportunities for active transport and delivery of antibiotics into the cytoplasm. In this regard, the siderophore-mediated antibiotic delivery was fruitfully exploited to promote the transport of oxazolidinones through the bacterial outer membrane. Inspired by the Trojan horse approach, Seiple and coworkers recently proposed a siderophore−antibiotic conjugate (SAC) to overcome the inability of Eperezolid-NH_2_ to diffuse through the cellular membranes. The antibiotic was connected to the bis-catecholate-based siderophore via the peptide WSPKYM to obtain a cleavable SAC. The inhibition of bacterial growth required the enzymatic cleavage of the linker by proteases, whereas the intact conjugate was inactive [60]. 

**Figure 2:**
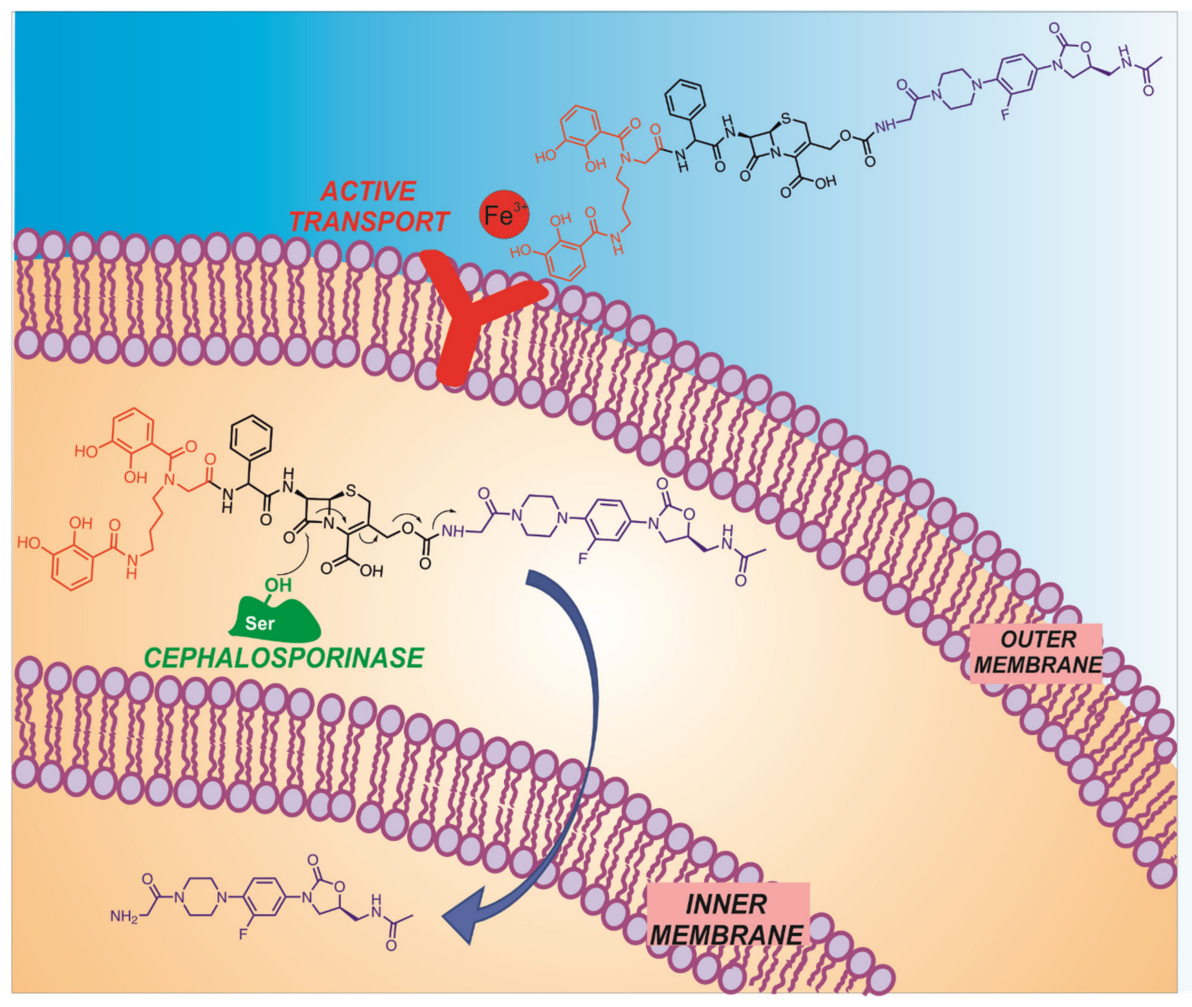
Sketched view of the cephalosporine-triggered release of oxazolidinone from the siderophore–cephalosporine–Eperezolid conjugate [61].

Moreover, the conjugation of Eperezolid with a siderophore linked to a cephalosporin was exploited to obtain a dual drug conjugate able to guarantee efficient passage of these drugs through the bacterial outer membrane (Figure 2). The proposed Trojan horse antibiotic, incorporating a cephalosporin linker, which will be hydrolyzed by bacterial β-lactamases, would allow an efficient release of Eperezolid inside the bacterial cell, so it can reach its intracellular ribosomal target. The enhanced concentration of the delivered oxazolidinone might also help to minimize the effects of efflux pumps, allowing the drug to exert its lethal effect on targeted GNB [61]. 

## 5. Perspectives, Challenges and Conclusions

The treatment of bacterial infections due to Gram-positive and Gram-negative strains is still challenging, especially with regard to resistant strains. The abuse of antibiotics has resulted in the increase of widespread antimicrobial resistance to commonly prescribed drugs, requiring the development of new active compounds able to manage severe microbial infection, reducing morbidity and mortality. 

The scenario described in this review shows the leading position of oxazolidinone antibiotics against multidrug-resistant GPB and drug-resistant Mtb, although until now only LNZ and TZL have been licensed for human use. Several oxazolidinone derivatives failed in the Phase I or Phase II clinical stages due to inherent toxicity and side effects. Moreover, the spectrum of activity of LNZ is relatively narrow and mainly covers GPB. 

The development of new oxazolidinone antibiotics with potent activity, a wide spectrum of activity and minimal adverse effects should be considered a priority, especially for the treatment of drug-resistant Mtb, since severe toxic effects are associated with its long-term treatment protocol. Currently, approximately 2 billion people are infected with TB and the arsenal against TB, the second most deadly infectious disease on the planet, is limited [62]. 

Nanotechnology tools could be decisive for the development of new oxazolidinone therapeutics since different antimicrobial agents could be co-entrapped and delivered by engineered nanoplatforms achieving a synergistic action, a broadening of the antibacterial spectrum and improved safety profiles compared to free drugs. While nanomedicine strategies are in use for the design of vaccine carriers [63], nanotechnological approaches to tackle drug-resistant infection diseases are still confined to the laboratory. Different nanotechnological platforms based on biocompatible materials such as hyaluronic acids [64,65], polyester (i.e., PLA) [66] and cyclodextrins [67] could be used for the development of antimicrobial nanomedicines [68,69]. 

The interactions among oxazolidinone antibiotics and metal ions are a completely unexplored topic. Metal complexation is known to affect the action of many drugs [70,71] and it plays a vital role in a vast number of biological processes contributing to the health or toxicity of the organism. As is known, the antibiotic interaction in vivo with several metal cations influences their bioavailability. For this reason, speciation studies, in terms of interactions of antibiotics with metal cations having relevant biological roles, are crucial [72,73]. 

The analytical determinations of oxazolidinone antibiotics in the biological matrices or as pollution analytes have been marginally investigated in the past and no critical discussion has been reported. Certainly, many aspects still need to be investigated regarding the analytical methods. First of all, the development of methodologies (or the validation of those already reported) for oxazolidinones other than LNZ. The second aspect concerns the matrix, since most of the studies were directed at the determination of LNZ in biological fluids or pharmaceutical preparations. Only recently did de Barros and coworkers [74] report a method for the simultaneous determination of LNZ among other drugs in the surface water of a Brazilian river, which is therefore useful for the determination in environmental field. To our knowledge, this paper is the only one reporting the determination of LNZ in natural waters. Considering the wide interest of these compounds from the environmental point of view, this aspect still needs further investigation. 

## Figures and Tables

**Table 1:** The main representative oxazolidine antibiotics licensed or under development.

Drug Name	Features	Ref.
Sutezolid (NU-100480)Pfizer	Phase II of clinical trials was completed. The results showed that the drug was well tolerated and safe.	[28,29]
Contezolid (MRX-1)	Phase III of clinical trials was completed in China. It is awaiting approval for the oral treatment of ABSSSI caused by GPB.	[30]
Radezolid (RX-1741)	Phase III clinical trials for the treatment of multidrug-resistant infections, including infections caused by LNZ-resistant strains. It is 11-times more active in comparison to LNZ. Currently, its safety profile has not been established and its advantages over LNZ and TDZ are not clear.	[14,31]
Delpazolid (LCB01-0371)	Phase I/Phase II of clinical trials are ongoing. The safety profile could be suitable for long-term therapies (i.e., TB). LegoChem Biosciences entered into a license agreement with RMX Biopharma for the development of Delpazolid in China. In addition, Delpazolid received an FDA orphan drug designation.	[32]
Posizolid (AZD2563/AZD5847) Astrazeneca	Phase II clinical trials discontinued. The results are not conclusive since the studies for the treatment of TB were discontinued.	[29]
TBI-223	It is under phase I clinical trial (NCT03758612) with the aim to evaluate its safety, tolerability and pharmacokinetics.	[28,33,34]
Tedizolid (TZD) formerly Torezolid	TZD is approved for the treatment of acute bacterial skin and soft tissue infections by the FDA and EMA. Compared to LNZ, TZD is significantly less expensive. Oral and intravenous formulations are available. Tedizolid phosphate is an orally absorbed phosphate prodrug of TZD. Tedizolid phosphate was the second oxazolidinone drug approved by FDA for the treatment of MRSA skin infections in 2014.	[35,36]

**Table 2:** Performance of different instrumental techniques for oxazolidinone detection.

Instrumental Techniques	Oxazolidinone	Matrices	LOD	Linear Concentration Range	Ref.
HPLC-UV	LNZ	Biological fluids	0.007 ≤ LOD ≤ 0.5 μg/mL		[37]
HPLC/UPLC-MS/MS	LNZ	Biological fluids	0.05 ≤ LOD ≤ 0.1 μg/mL		[37]
HPLC-MS/MS	OTB-658	Monkey blood	0.74 ng/mL	10–2000 ng/mL	[38]
UPLC-MS/MS	TZD	Plasma	10 ng/mL *	0.74–1500 ng/mL	[40]
	TZD	Humor of rabbit	1.97 ng/mL	4.98–1000 ng/mL	[39]
Spectrofluorimetry	LNZ	Pharmaceutical formulations	110 ng/mL	0.5–50 μg/mL	[41]
	LNZ	Pharmaceutical formulations	4.28 ng/mL	20–400 ng/mL	[42]
Voltammetry	LNZ	Pharmaceutical formulations	50 ng/mL	up to 200 μg/mL	[45]
	LNZ	Pharmaceutical formulations	11 ng/mL	0.084–10.5 μg/mL	[47]
	LNZ	Plasma	0.98 ng/mL	0.0085–2.70 μg/mL	[43]
	LNZ	Pharmaceutical and biological samples	0.47 ng/mL	0.01–0.2 μg/mL	[44]
	LNZ	Cationic surfactant media	50 ng/mL	0.25–6.41 μg/mL	[21]

* LOQ.

## Data Availability

Not applicable.
